# Eco-efficient materials for agricultural crops based on a mineral rich in MOR- and HEU-type zeolites

**DOI:** 10.3762/bjnano.17.26

**Published:** 2026-02-26

**Authors:** Esperanza Yamile de la Nuez-Pantoja, Inocente Rodríguez-Iznaga, Gerardo Rodríguez-Fuentes, Vitalii Petranovskii, Ariel Martínez García, José Juan Calvino Gámez, Daniel Goma Jiménez

**Affiliations:** 1 Instituto de Ciencia y Tecnología de Materiales (IMRE), Universidad de La Habana, Zapata y G, 10400, La Habana, Cuba; 2 Centro de Nanociencias y Nanotecnología (CNyN), Universidad Nacional Autónoma de México (UNAM), 22860, Ensenada, B.C., México; 3 Departamento de Ciencia de los Materiales, Ingeniería Metalúrgica y Química Inorgánica, Facultad de Ciencias, Universidad de Cádiz, Campus Río San Pedro, 11510 Puerto Real, Cádiz, España

**Keywords:** adsorption, agroecological fertilizer, ion exchange, MOR–HEU zeolite mixture, natural zeolite

## Abstract

Natural zeolites have great potential as nutrient carriers to develop eco-efficient materials for massive use in agriculture. Zeolitic minerals usually contain only one dominant zeolite type. The use of minerals with mixtures of zeolites in similar proportions can affect the interaction of chemical species with the zeolitic matrix, altering the behaviour of the resulting materials. In this work, a mineral consisting mainly of a mixture of two zeolites, mordenite (MOR) and clinoptilolite-heulandite (HEU) with equivalent fractions, was used to develop materials carrying nutrients (N, P, and K) for agricultural crops. The mineral matrix provides important elements such as K and Si, while N and P were incorporated into the material by treatment with ammonium hydrogenphosphate and urea. The presence of superficially adsorbed PO_4_^3−^, NH_4_^+^ exchanged in zeolites, and urea arranged on the surface so that it covers the material and interacts with the zeolitic frameworks, was evidenced by Fourier-transform IR spectroscopy, adsorption measurements, scanning electron microscopy, scanning transmission electron microscopy, and other methods, as well as through culture tests. The complexity of the multiphase zeolitic support leads to changes in the position and intensity of FTIR bands compared to other similar materials developed using simpler zeolitic carriers dominated by HEU zeolite. The most intense NH_4_^+^ band was observed at 1402 cm^−1^, while for a HEU zeolite it was at 1540 cm^−1^. This difference was associated with a higher NH_4_^+^ content in MOR compared to HEU. Accordingly, the shift experienced by the urea amino group bands when it interacts with the frameworks of these zeolites is different. The applied treatments did not affect the structures (as evidenced by XRD) and other qualities of these zeolites, highlighting their ion-exchange and adsorption properties for nutrient release and reversible water retention. This is essential for the use of this material as a slow-release fertilizer that efficiently provides nutrients for the agroecological development of plants, as evidenced in the cultivation tests.

## Introduction

Traditional chemical fertilizers, widely used in agriculture, undergo significant nutrient losses resulting from dissolution and consequent drag and infiltration under the action of irrigation water and rain [1]. This problem, which is also associated with volatilization and emission of NO, NH_3_, and other gases into the atmosphere, poses severe risks to the environment and causes serious damage to human health [2]. Minerals rich in natural zeolites are a viable alternative for the development of efficient, agroecologically sustainable, and low-cost fertilizer materials for massive applications [3–4].

Natural zeolites are porous crystalline hydrated aluminosilicates. They have a three-dimensional, rigid, negatively charged structure formed by silicon and aluminium tetrahedra connected through their vertices by bridging oxygen atoms. This arrangement forms a network of interconnected channels and cavities with a large surface area, where water molecules and mobile cations (Na^+^, K^+^, Ca^2+^, Mg^2+^) are located to neutralize the excess negative charge of the structure. As a result, these materials have important intrinsic properties such as ion exchange and adsorption [5]. These qualities allow them to retain and carry chemical species of agricultural interest, such as PO_4_^3−^, NH_4_^+^, NO_3_^−^, and molecular compounds (CO(NH_2_)_2_) minimizing losses during dissolution [6–9]; also, more than 30% of their total weight is water, which provides additional moisture during hot and dry periods. These characteristics make zeolites potentially applicable in agriculture for the development of modern fertilizers with controlled release and increased nutrient utilization efficiency [3–4]. Several studies report improved growth, yield, and protection against pests and diseases in a wide range of agronomic and horticultural crops when zeolitic minerals are applied [10–11]. Also, native cations present in zeolites (Na^+^, K^+^, Ca^2+^, Mg^2+^) can be utilized by crop plants during their development. Furthermore, the silicon contained in its matrix is considered an important element due to its role in the physiology and biochemistry of agricultural crops [12].

The cation exchange and adsorption properties of zeolites form the basis for the modification of zeolitic materials developed by our working group. These properties enabled the development of a technological procedure described in [13–14], which can enrich natural minerals with essential additional nutrients without significantly affecting their original cationic composition and without producing environmentally harmful chemical residues. This procedure has made it possible to develop environmentally friendly zeolitic substrates and slow-release fertilizers as an agroecological alternative for improving plant development. These products have a low nutrient content compared to conventional fertilizers; to achieve beneficial results, they provide the minimum amounts necessary for balanced crop growth.

Several widely available types of natural zeolites have been used in the development of these modern and economical fertilizer materials [2–3,6,8]. Among all the zeolites used, clinoptilolite (CLI) and mordenite (MOR) stand out. Published studies have outlined that the level of development achieved is influenced by the properties of the specific zeolite type used, which is valid both for the development of materials and for their use [15–17]. The nature of the zeolite matrix plays a key role in its interaction with various chemical species, which affects the behaviour of the resulting material.

In general, due to the peculiarities of geochemical processes, only one main zeolitic phase is usually formed during the formation of zeolite deposits. This main phase coexists with other minor phases, such as other types of zeolite, quartz, and feldspar; accordingly, zeolitic minerals with mixtures of zeolites in similar proportions are rare. However, the use of local zeolite deposits, even with a complex mixture of phases, appears to be economically feasible. First of all, natural zeolites, unlike synthetic analogues, do not require expensive chemical raw materials and energy-intensive stages of hydrothermal synthesis. The main costs of their exploitation are associated with extraction and mechanical processing, which significantly reduces the cost of the final product. At the same time, even multicomponent zeolite-containing rocks (e.g., clinoptilolite, heulandite, mordenite, quartz, and plagioclase) retain high sorption and ion exchange activity, sufficient for a wide range of applications.

Local extraction minimizes transportation costs, which is especially important for regions with significant natural reserves of zeolite-containing rocks. In addition, the involvement of such deposits in economic turnover contributes to the development of local industrial infrastructure and increases added value at the regional level. Even with a heterogeneous mineral composition, it is possible to create products with target properties by optimizing modification processes.

FTIR studies on NH_4_^+^ ions supported on various zeolites have shown changes in the intensity and position of ammonium bands. The literature [18–19] suggests that there are two frequency regions associated with the bending vibration of ammonium ions, namely, a triplet with intense peaks in the 1350–1550 cm^−1^ range and a less intense doublet in the 1600–1800 cm^−1^ range, attributed to different types of hydrogen-bonded NH_4_^+^ complexes. Studies [20–21] of natural CLI from the Caimanes deposit, Cuba, and its nickel form (Ni^2+^-CLI) treated with ammonium solutions showed FTIR bands at about 1400 and 1443 cm^−1^, respectively, associated with the N–H bond bending vibration attributed to the NH_4_^+^ ion. Yadav et al. [22] studied natural CLI from the Amazon (Dolphin brand, FM-906) using FTIR and reported a high-intensity band around 1358 cm^−1^ associated with the deformation vibration of ammonium. De la Nuez Pantoja et al. [15] detected in the FTIR spectrum of natural clinoptilolite-heulandite (HEU) from the Tasajeras deposit (Cuba), exchanged with ammonium hydrogenhosphate ((NH_4_)_2_HPO_4_, DAP), the presence of a triplet of adsorption bands around 1403, 1450, and 1540 cm^−1^ and a less intense band around 1691 cm^−1^. All these bands were associated with N–H bending modes of NH_4_^+^, with different bonding interactions, with the most intense band measured at 1540 cm^−1^. Similar results were obtained by Adriano et al. [23] and Xu et al. [24] in their studies on a NH_4_^+^-MOR, which showed a broad and intense band around 1400 cm^−1^ assigned to the NH bond of ammonium. Similarly, Wei et al. [25] observed two strong adsorption bands at 1404 and 1440 cm^−1^ in the spectra for an NH_4_^+^-exchanged sodium MOR, associated with N–H bending modes of NH_4_^+^ arrangements with different bond interactions. Bonelli et al. [26] reported for an NH_4_^+^-ZSM-5 zeolite the presence of a triplet of overlapping FTIR bands in the range of 1350–1550 cm^−1^ (1405, 1465, and 1500 cm^−1^). For analcime undergoing NH_4_^+^ exchange, the triplet appears as two weak shoulders around 1427 and 1442 cm^−1^ and a strong adsorption band at 1468 cm^−1^ [27]. For natural NH_4_^+^-chabazite, a strong IR band at 1465 cm^−1^ and a weak shoulder near 1407 cm^−1^ have been reported [28]. FTIR spectra of NH_4_^+^-SAPO-34, reported by Liu et al. [29], exposed the formation of bidentate and tridentate NH_4_^+^ structures stabilized inside the cages and channels of the framework of this material; a new band around 1400 cm^−1^ corresponding to the bending vibration of NH_4_^+^ at Brønsted acid sites was detected. This is consistent with the results obtained by Putra et al. [30] and Zecchina et al. [18], which show absorption bands near to 1400 cm^−1^ corresponding to the bending vibrations of N–H bonds in NH_4_^+^-modified natural zeolites.

In general, when ammonium interacts with the oxygen atoms of the zeolitic structure, various configurations are formed, each of which exhibits different interaction strength depending on the type of species present. These features affect the rate at which cations are delivered to the external environment. This aspect is relevant for agricultural applications, where zeolites are used as controlled-release fertilizers [28] for a range of nutrients. Besides this, most prior work has focused on zeolitic minerals with HEU-dominant systems, making those with MOR/HEU mix a distinct and less studied case.

This work presents a study on the zeolitic mineral CLIM from the San Andrés deposit (Cuba), consisting mainly of a mixture of two zeolites, MOR and HEU, in approximately equal proportions. As mentioned above, the use of local zeolite-containing deposits appears to be economically feasible, despite the complex mixture of phases present. The extracted zeolite ores were modified with solutions of DAP and urea to obtain materials containing both carrying essential nutrients (N, P, K) and other elements (Si) important for agricultural crops. Particular attention was paid to the analysis of the interaction of nitrogen and phosphorus species on this complex multiphase zeolitic carrier, applying Fourier-transform infrared spectroscopy (FTIR), X-ray diffraction (XRD), scanning electron microscopy (SEM), scanning transmission electron microscopy (STEM), N_2_ physisorption, and other research methods.

## Results and Discussion

### Characterization of DAP-modified zeolite CLIM

The chemical compositions of natural zeolite (CLIM) from the San Andrés deposit and its modified forms (CLIM_f_) with ammonium hydrogenphosphate are presented in Table 1. These data show that, in CLIM, the main cation components are Ca^2+^ and K^+^, in this order. The potassium content stands out compared to other zeolitic minerals, which is important because the native element K, as well as P and N incorporated in the modified samples (CLIM_f_) are essential macronutrients for agricultural crops [31]. CLIM also contains large amounts of other beneficial elements, such as Si, whose positive effect on crops and soil is widely recognized [12].

**Table 1:** Chemical composition (wt %) of natural zeolite (CLIM) and its modified forms (CLIM_f_) expressed in elemental form. The numbers after “f” in the samples labels indicate the percentage of fertilizer (DAP) applied to CLIM in order to obtain CLIM_f_.

Elements	Samples			

	CLIM	CLIM_f2_	CLIM_f3.5_	CLIM_f7_

Si	28.53 ± 1.23	27.98 ± 1.19	27.57 ± 1.19	26.67 ± 1.19
Al	6.19 ± 6 × 10^−2^	6.08 ± 6 × 10^−2^	5.99 ± 6 × 10^−2^	5.79 ± 6 × 10^−2^
Ca	2.74 ± 1 × 10^−3^	2.68 ± 1 × 10^−3^	2.64 ± 1 × 10^−3^	2.56 ± 1 × 10^−3^
Mg	0.39 ± 4 × 10^−4^	0.38 ± 4 × 10^−4^	0.37 ± 4 × 10^−4^	0.36 ± 4 × 10^−4^
Fe	1.40 ± 3 × 10^−4^	1.37 ± 3 × 10^−4^	1.35 ± 3 × 10^−4^	1.31 ± 3 × 10^−4^
Na	0.99 ± 2 × 10^−3^	0.97 ± 1 × 10^−3^	0.96 ± 1 × 10^−3^	0.93 ± 1 × 10^−3^
K	1.86 ± 5 × 10^−4^	1.83 ± 6 × 10^−4^	1.79 ± 6 × 10^−4^	1.73 ± 6 × 10^−4^
P	–	0.39 ± 1 × 10^−4^	0.68 ± 2 × 10^−3^	1.31 ± 4 × 10^−3^
N	–	0.28 ± 1 × 10^−2^	0.47 ± 1 × 10^−2^	0.92 ± 2 × 10^−2^

### FTIR studies of natural zeolite modified with DAP

**Figure 1:**
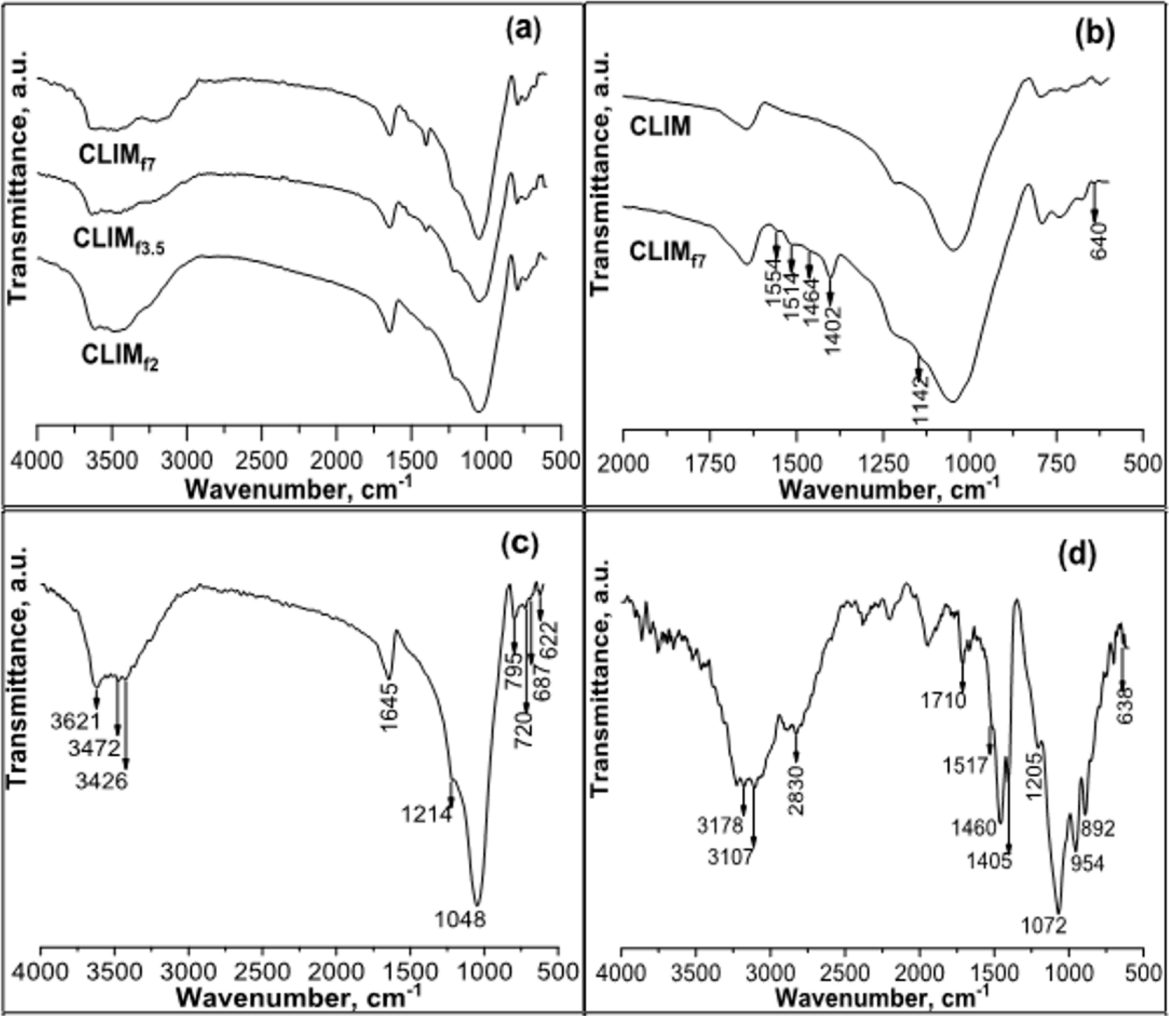
FTIR spectra of modified samples (CLIM_f_) and raw material (CLIM). (a) CLIM_f_ obtained as a result of modification using different amounts of DAP (2.0%, 3.5% and 7.0% corresponding to CLIM_f2_, CLIM_f3.5_ and CLIM_f7_, respectively). (b) Magnification in the range from 600 to 2000 cm^−1^ for spectrum “CLIM_f7_” of Figure 1a compared to CLIM. (c) CLIM. (d) DAP.

The FTIR spectra (Figure 1) of CLIM and CLIM_f_ (CLIM modified with DAP) samples show characteristic zeolite bands in the 620–1214 cm^−1^ region, which are associated with the silicon and aluminium tetrahedral groups (SiO_4_ and AlO_4_) of the crystal lattice of MOR and HEU. The bands in the 3400–3600 cm^−1^ region are attributed to water molecules coordinated with native charge-compensating cations (Na^+^, K^+^, Ca^2+^, Mg^2+^) and the stretching of OH groups, while those observed at 1645 cm^−1^ are associated with deformation vibrations related to water [32–33].

The changes obtained as a result of CLIM modifications with DAP (Figure 1a,b) are best visualized in the expanded view shown in Figure 1b, a magnification in the range of 600–2000 cm^−1^ corresponding to the modified material with a higher DAP content (7.0%). New bands in the modified samples (CLIM_f_), observed at 1402, 1464, 1514, and 1554 cm^−1^ are related to the N–H bond bending mode, associated with NH_4_^+^ species [18–19,24], while the bands visualized at 640 and 1142 cm^−1^ are associated with the asymmetric stretching and bending modes, respectively, of the P-O bonds in PO_4_^3−^ anions [34–35]. Both ions, NH_4_^+^ and PO_4_^3−^, were the result of modifications with DAP. It is important to note that the intensity of these bands increases proportionally to the content of the incorporated DAP and that they undergo changes in shape and positional shift with respect to the DAP spectrum (Figure 1d), which is associated with the interaction of NH_4_^+^ and PO_4_^3−^ ions with the zeolitic support.

Zechina et al. [18] and Bučko et al. [19] studied the infrared spectra of some zeolites modified with ammonium and concluded that the IR spectrum of NH_4_^+^-zeolite is very complex since the vibrational characteristics of the incorporated species are influenced by their interaction with the oxygen atoms of the zeolitic lattice, which, in turn, depends on their position within the zeolitic mineral.

NH_4_^+^ species on numerous zeolites have similar FTIR profiles; in particular, they are characterized by a bending triplet, with intense peaks in the range of 1350–1550 cm^−1^, attributed to different types of hydrogen-bonded NH_4_^+^ complexes, indicating the presence of bidentate and tridentate coordination modes [18,26,29,36]. The monodentate configuration has one hydrogen atom, out of four, that interacts with one oxygen atom of the framework; the bidentate configuration has two hydrogen atoms interacting with two different oxygen atoms of the framework, and so on [18–19]. It is the number of these bonds that affects their stability [19,34]. NH_4_^+^ interacts with Brønsted acid sites, acting as a hydrogen bond that anchors the NH_4_^+^ ions to oxygen atoms in zeolite, and forms coordinated bonds only when interacting with terminal silanol groups [19].

For industrial applications, it is important to understand the environment surrounding NH_4_^+^ ions within zeolite cavities. In our case, according to the literature [18,27] and FTIR results, the interaction between ammonium molecules and the MOR structure allows for the formation of bidentate and tridentate species. Studies carried out by Gualtieri et al. [28] led to the conclusion that, in bidentate structures, hydrogen bonds exhibit weak interaction with the oxygen atoms of the structure; this means that the ammonium molecules interact weakly with the zeolitic lattice and, as a result, can easily exchange with the external medium. In contrast, in tridentate structures, hydrogen bonds are more strongly linked with the oxygen atoms of the structure; therefore, the ammonium molecules interact more strongly with the zeolitic support and are more difficult to exchange with the medium. This behaviour is of great importance for agronomic applications [27] as it allows NH_4_^+^ ions to gradually exchange with the environment. This ensures that there are no significant losses of this element as in the case of a single application of water-soluble fertilizers, which enables plants to use it for a long time.

Overall, these results are close to those expected for natural zeolite modified with these chemical species. However, there are differences with respect to other NH_4_-zeolites, such as the HEU from the Tasajeras deposit, Cuba [15], also modified with DAP. Thus, after modification with DAP, the most intense ammonium band is revealed at 1402 cm^−1^ in this natural zeolite (CLIM), whereas for the natural zeolite of the HEU type from the Tasajeras deposit [15], the most intense band is at 1540 cm^−1^. The characteristics of this vibrational band for the CLIM_f7_ sample are similar to those found in MORs exchanged with ammonium, including natural [23] and synthetic [24–25] ones.

These results may be related to the higher NH_4_^+^ content in the cation exchange sites in the MOR channels contained in the San Andres zeolitic mineral. In this natural zeolite (CLIM), the MOR phase content is much higher than in the natural zeolite from the Tasajeras deposit, composed mainly of HEU. There are differences at the structural level between the two types of zeolites, MOR and HEU. MOR has channels with a higher number of tetrahedra in the channel cross section and larger dimensions than HEU, which leads to differences in their structural charge. The largest channel of HEU has ten tetrahedra and a size of 3.1 × 7.5 Å [16,35], while, in MOR it has twelve tetrahedra with a size of 6.7 × 7.0 Å [37]. Therefore, this observed difference may be the result of differences in the interaction of ammonium with the structure of each type of zeolite.

Considering the properties of the ions involved in the exchange, it can be assumed that the incorporation of NH_4_^+^ occurs mainly through ion exchange processes (Equation 1) with the native cations of these zeolites (MOR and HEU).


[1] $${\text{M}}^{n+}{\text{CLIM}}_{\left(\text{s}\right)}\;+\;{\text{nNH}}_{4\;\left(\text{aq}\right)}^{\;+}\;==\;{\left({\text{NH}}_{4}^{\;+}\right)}_{n}\text{​}{\text{CLIM}}_{\left(\text{s}\right)}\;+\;{\text{M}}_{\;\left(\text{aq}\right)}^{n+}$$

where CLIM is the zeolitic phase (a mixture of HEU and MOR) and M*^n^*^+^ are their natural cations (Ca^2+^, Na^+^, K^+^, Mg^2+^). The subscripts (s) and (aq) denote “in zeolite” and “in solution,” respectively; *n* can take integer values from 1 to 2.

In contrast, phosphorus is superficially retained in the form of PO_4_^3−^ (hydrated ionic radius of 3.39 Å) [38] by adsorption and occlusion processes in the porosity of the zeolitic support, compensating for its charge with cations (K^+^, Ca^2+^, etc.) exchanged from zeolitic phases and some amount of unexchanged ammonium ions. According to published data [21], phosphate anions can form part of an electric double layer with concurrent cations (Na^+^, Ca^2+^, NH_4_^+^, etc.) bound by electrostatic attraction forces.

Natural zeolite from the San Andres deposit is rich in mixed zeolite phases of the HEU and MOR type, whose crystal structures are formed by interconnected channel systems in which cations are located to compensate for the structural charge. HEU is formed by three channels, named A, B and C, with dimensions of 3.1 × 7.5, 3.6 × 4.6 and 2.8 × 4.7 Å, respectively [16,35]. For MOR, three types of channels are described, distinguished as main, secondary, and lateral (“side pocket”) with dimensions of 6.7 × 7.0, 2.6 × 5.7 and 3.4 × 4.8 Å [37], respectively. In accordance with these dimensions, the NH_4_^+^ cation (hydrated ionic radius of 3.31 Å) [21] can diffuse through these zeolitic channels for exchange (Equation 1), which occurs mainly with Ca^2+^ and K^+^. In addition, it should be expected that the intracrystalline diffusion of ammonium ions is favoured in MOR since it has channels with a larger diameter. It has been reported that, in MOR zeolites, Ca^2+^ and K^+^ cations are located in smaller channels (secondary and lateral) [37]. In the case of HEU, it has been reported that K^+^ is located in channel C, while Ca^2+^ is located in channels A and B, predominantly in the latter [16,35]. Therefore, it should be expected that the exchanged NH_4_^+^ will be located, at least temporarily, in the secondary and lateral channels of MOR, and in the three channels of HEU.

### FTIR study of CLIM consecutively modified with DAP and urea

**Figure 2:**
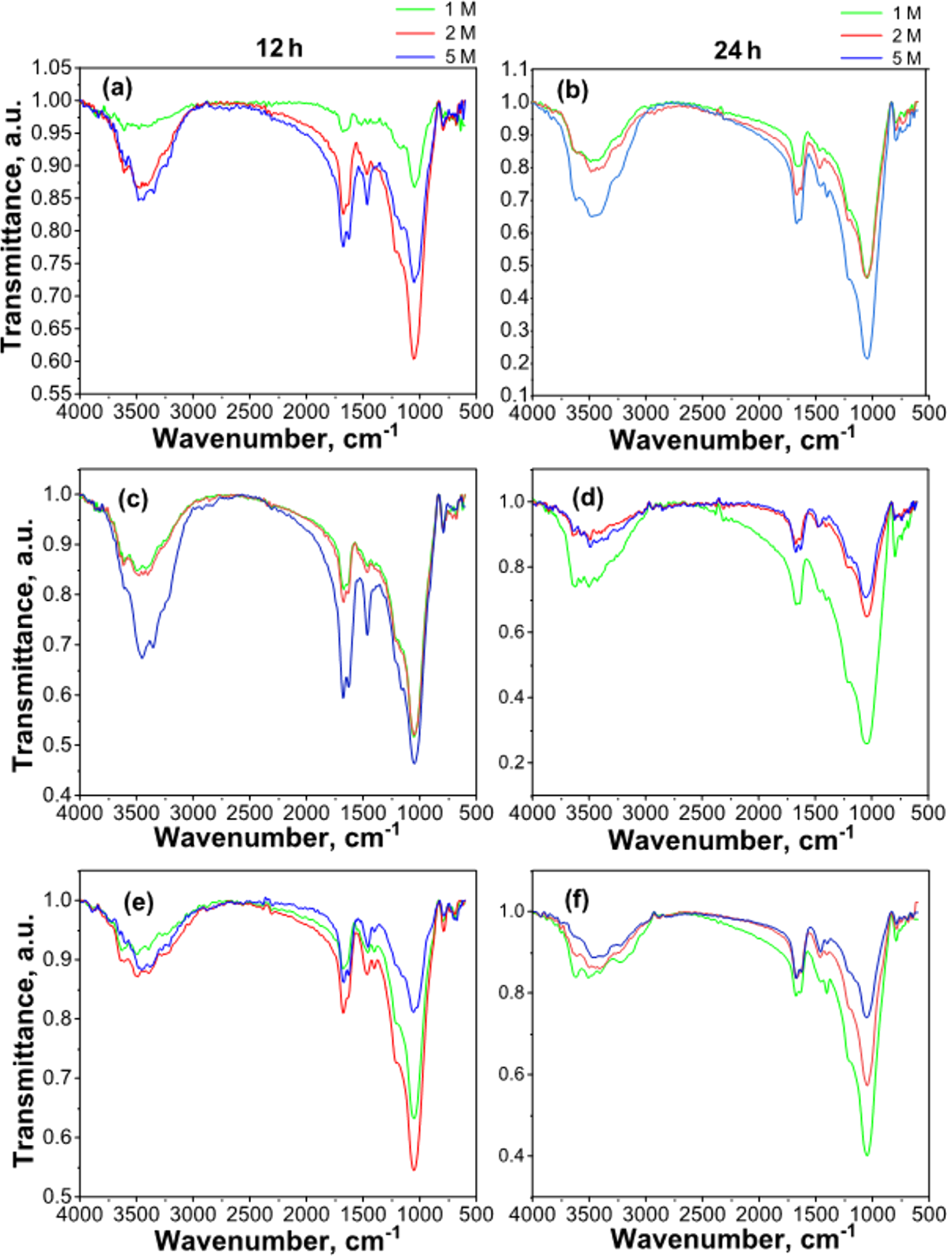
FTIR spectra of CLIM_f-U_ materials obtained from CLIM consecutively modified with DAP and urea solutions at different concentrations (1, 2, and 5 M) for contact times of 12 and 24 h: (a, b) with 2% DAP, (c, d) with 3.5% DAP, and (e, f) with 7% DAP.

**Figure 3:**
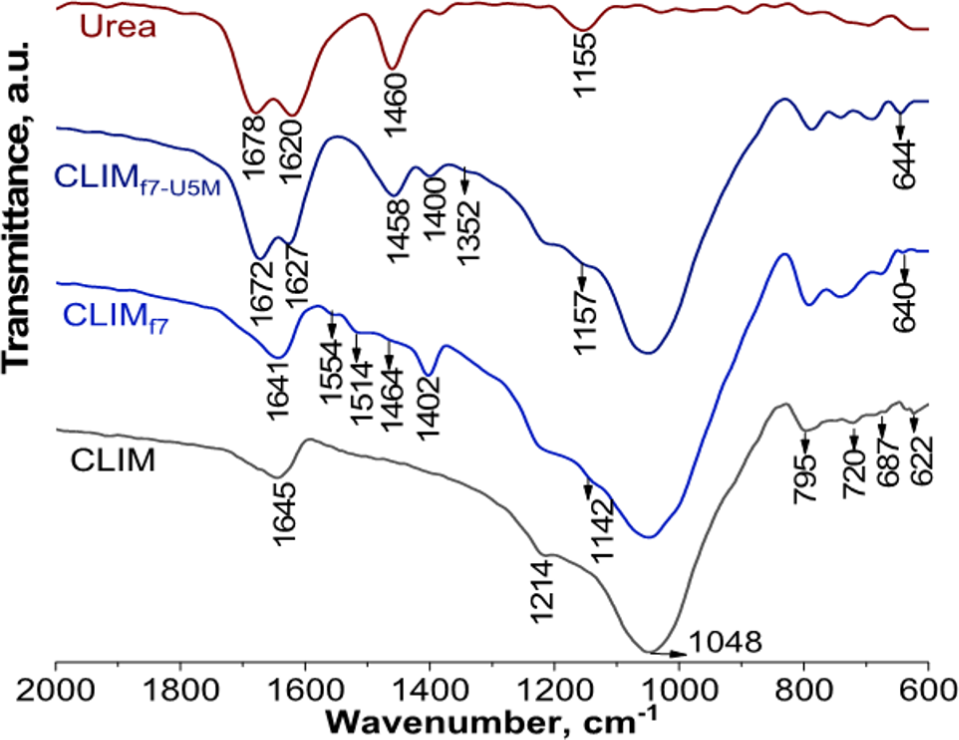
Enlargement of the FTIR spectrum of the CLIM_f7-U5M_ material showing differences resulting from successive modifications.

Figure 2 and Figure 3 show the FTIR spectra of CLIM_f-U_ materials obtained from CLIM sequentially modified with DAP and urea solutions.

Along with the bands associated with ammonium and phosphate, the spectra (Figure 2) showed the presence of bands characteristic of urea, whose intensities increase with the concentration of the modifying solutions. There are differences in the position of these bands with respect to their location in urea, which is in agreement with the data reported in [9,15,39].

The spectral region between 600 and 2000 cm^−1^ (Figure 3) for the material with a higher DAP and urea content allows for better visualization of the bands and the resulting differences. The characteristic symmetric deformation of the amino group, located at 1678 cm^−1^ in urea, undergoes a shift towards lower frequencies, 1672 cm^−1^ in CLIM_f7-U5M_, while the asymmetric deformation of the same group, located at 1620 cm^−1^ in urea, appears in CLIM_f7-U5M_ at a higher frequency (1627 cm^−1^). At around 1460 cm^−1^, a vibration associated with the asymmetric stretching of the C–N bond can be observed [9,39].

According to [9,15,39], the observed shifts in the urea bands are mainly associated with the weakening of the N–H bond of the amino group, probably due to interaction with the zeolitic structure via hydrogen bonding. These shifts were also observed with natural HEU from the Tasajeras deposit modified with urea [15]. However, there are differences, mainly in the vibrations associated with the asymmetric bending of the urea amino group. In the case of HEU from the Tasajeras deposit, a slightly larger displacement (12 cm^−1^) was observed compared to the 7 cm^−1^ shift observed for this natural zeolite (a mixture of MOR and HEU) from the San Andrés deposit under study.

Urea could have been located outside the material channels, adhering to the zeolitic surface [9,40]. Therefore, interaction at the surface level and coating of the mineral material surface can be assumed. Hydrogen bonds can be established through water molecules interacting directly and simultaneously with the zeolitic phase and urea, that is, water molecules in intermediate positions [9,39]. Given the similarity in size between urea molecules (5.6 × 6.3 × 3.0 Å) [41] and the maximum channel sizes of HEU (3.1 × 7.5 Å) and MOR (7.0 × 6.5 Å) [5,36–37], it is possible that urea can be retained in zeolitic channels [9,39], mainly in MOR with its channels of larger dimensions. Occluded urea should be retained more firmly than urea adsorbed in mesopores and on the outer surface of crystals; consequently, its release process will be slower and more limited [7,40].

It is important to note in the spectra (Figure 2 and Figure 3) the persistence of the bands associated with ammonium cations and phosphate anions. This result allows to conclude that treatment with urea solutions does not leach out the essential macronutrients nitrogen and phosphorus supported on the zeolitic support during the first stage of modification. This arrangement of urea on the surface of the material will modulate the delivery of nutrients to the soil solution, improving the characteristics of these materials and allowing for the delivery of nutrients to be controlled over time. In addition, it should be noted that if urea is hydrolysed to form NH_4_^+^ and NO_3_^−^, these ionic species can also be retained by exchange and adsorption on the zeolitic carrier for use by agricultural crops [15].

Based on the above, it can be concluded that these new zeolitic materials have great potential as agroecological developments. Studies on cultivation of corn in pots, which are a common diagnostic test for these purposes [15], confirm the aforesaid. The results obtained in these studies showed that the plants with the best response to the evaluated parameters (stem diameter, leaf area, and plant height) corresponded to the application of the materials prepared by sequential modification with DAP and urea solutions with a concentration of 5 M and a treatment time of 24 h. The difference in DAP content did not result in significant variation in the results obtained during these cultivation trials. For more detailed information on these experimental materials, studies were conducted using XRD, N_2_ adsorption isotherms, SEM, TEM, and STEM. However, they will only be presented for the most interesting materials, namely those that were modified with 2.0% and 3.5% DAP and then with a 5 M urea solution, that is, the materials CLIM_f2-U5M_ and CLIM_f3.5-U5M_, respectively. Detailed potted plant studies will also be presented for these materials.

Table 2 shows the nitrogen, phosphorus, and potassium contents in these two materials, which indicate a marked increase in total N content after treatment with urea (CLIM_f-U_). This aspect would avoid the limitations of other zeolitic products developed [13–14] in terms of nitrogen supply at the beginning of the growth cycle and controlled supply of nutrients. It should be noted that there is a slight decrease in P and K contents (Table 1 and Table 2), but these elements are not completely washed out of the zeolitic mineral. This fact is of great importance for agriculture since these elements, along with N, are essential for plant development and are needed by plants to a greater extent than other nutrients.

**Table 2:** Total nitrogen (N_t_), phosphorus (P), and potassium (K) contents and textural parameters determined by N_2_ adsorption isotherms at 77 K for materials obtained by successive treatments with DAP and urea (CLIM_f-U_).^a^

Materials	CLIM	CLIM_f2-U5M_	CLIM_f3.5-U5M_

N_t_	–	2.93 ± 3 × 10^−2^	3.61 ± 4 × 10^−2^
P	–	0.21 ± 6 × 10^−3^	0.53 ± 1 × 10^−2^
K	1.86 ± 5 × 10^−4^	1.77 ± 2.5 × 10^−4^	1.73 ± 2.5 × 10^−4^
A*s_B_* (m^2^·g^−1^)	80.28	32.63	41.28
A*s_t_* (m^2^·g^−1^)	24.30	22.28	18.70
*V_Microp_* (cm^3^·g^−1^)	0.026	0.0046	0.010

^a^A*s_B_*: surface area according to BET. A*s_t_*: external surface area calculated by t-curve. *V_Microp_*: micropore volume.

In general, it can be observed that successive modification with DAP and urea to obtain modified materials leads to a decrease in the value of these surface parameters. This is a result of the presence of nitrogen and phosphorus species adsorbed and occluded on the surface of these materials, as previously shown. This double treatment can be translated into a reduction in available space or surface area on this natural zeolite.

### X-ray diffraction analysis

**Figure 4:**
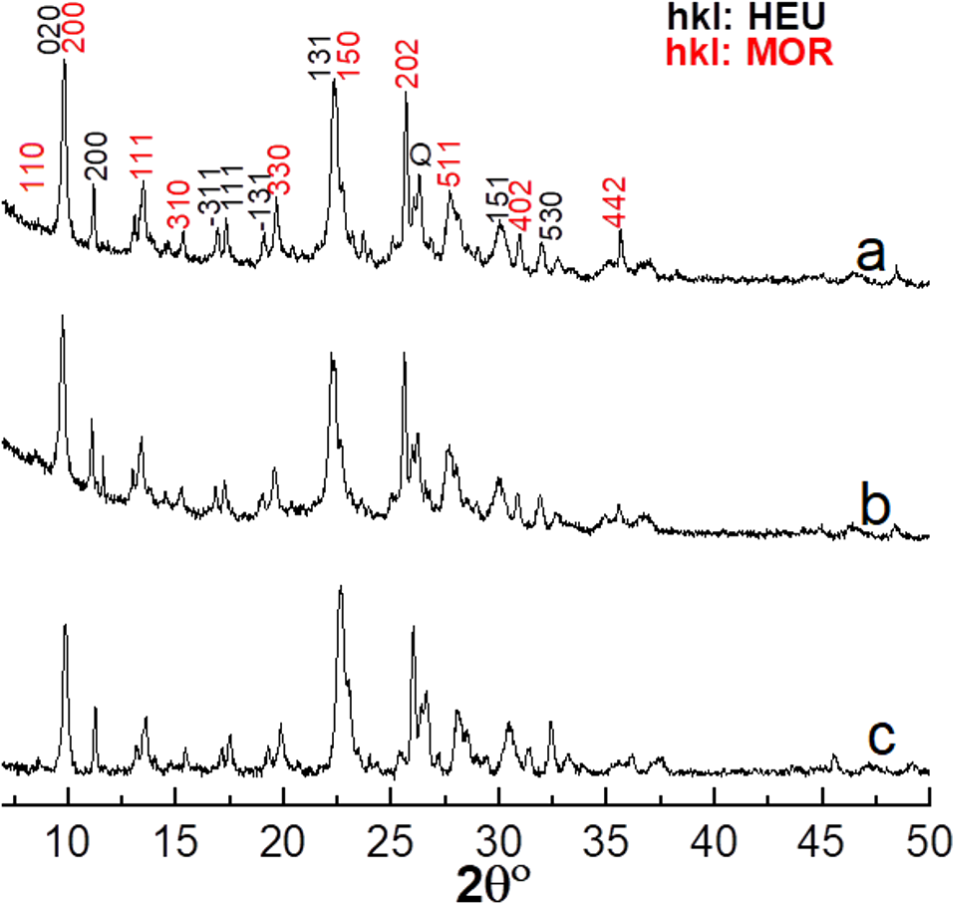
X-ray diffraction patterns of the CLIM (a), CLIM_f2-U5M_ (b) and CLIM_f3.5-U5M_ (c) samples. Miller indices (hkl) of the main diffraction planes of HEU (HEU) and MOR (MOR) are shown.

Figure 4 shows X-ray diffraction (XRD) patterns of the materials under study, which evidenced that this natural zeolite (CLIM) is mainly formed by a mixture of HEU (JCPDS Card 25-1349) and MOR (JCPDS Card 11-0155) with accompanying minority phases such as quartz (pdf No. 46-1045).

No indications of significant changes in the structure of these zeolites, caused by the processes of obtaining fertilizer materials, were revealed in the XRD patterns. The main differences are in the variation of the relative peak intensity as a consequence of the ion exchange processes (Equation 1) occurring during treatment of CLIM with DAP solution, which is in agreement with the data reported in [16,21,42].

### Scanning electron microscopy and scanning transmission electron microscopy

**Figure 5:**
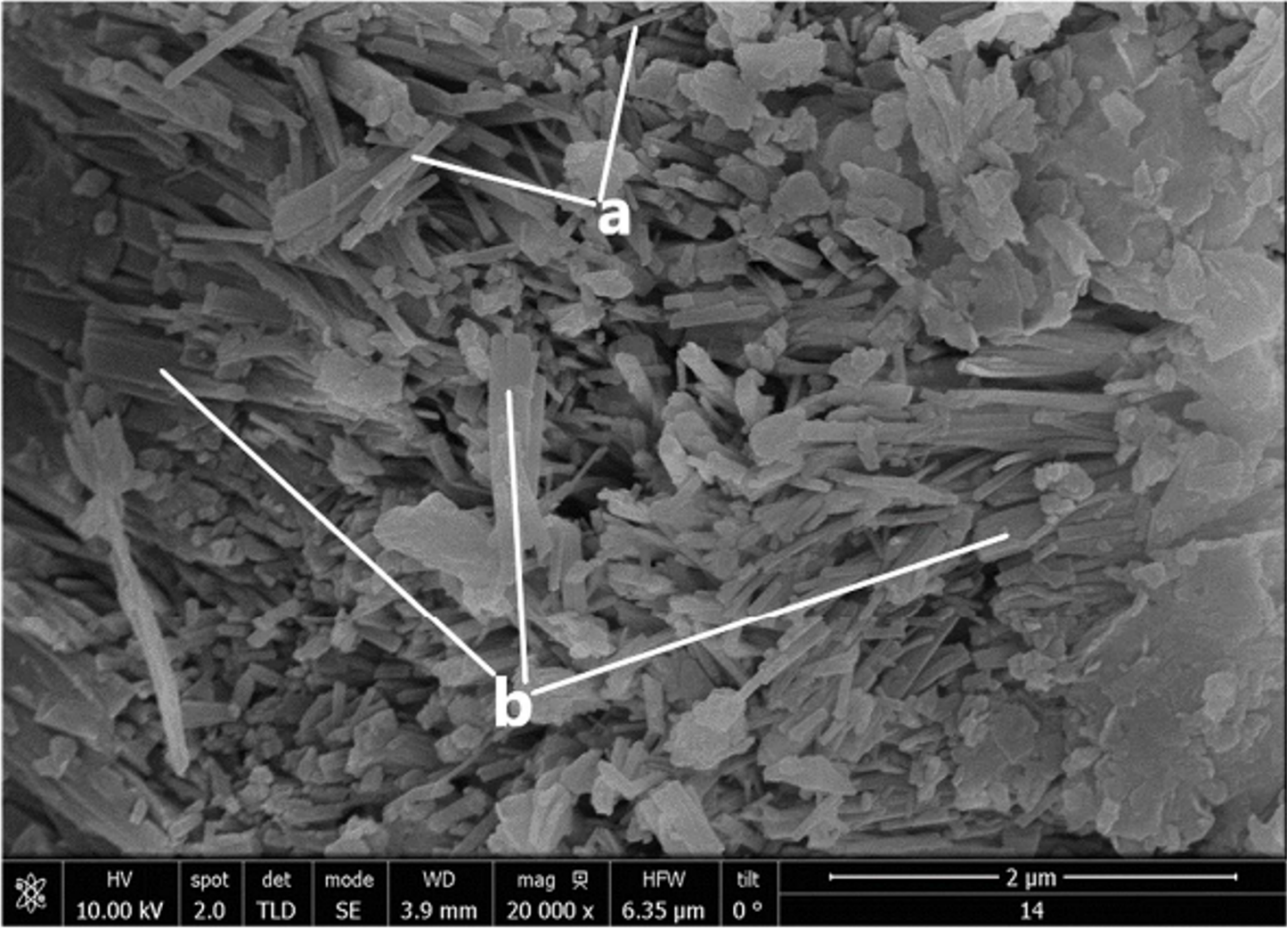
SEM micrograph of CLIM showing MOR crystals with a fibrous shape (a) and HEU crystals with a laminar habit (b).

Microphotography (Figure 5) of natural zeolite (CLIM) revealed an irregular surface with dispersed agglomerates and single particles with different morphologies, where the mineral nature with impurities affects the observation of surface details and obscures crystalline habits [43–44].

**Figure 6:**
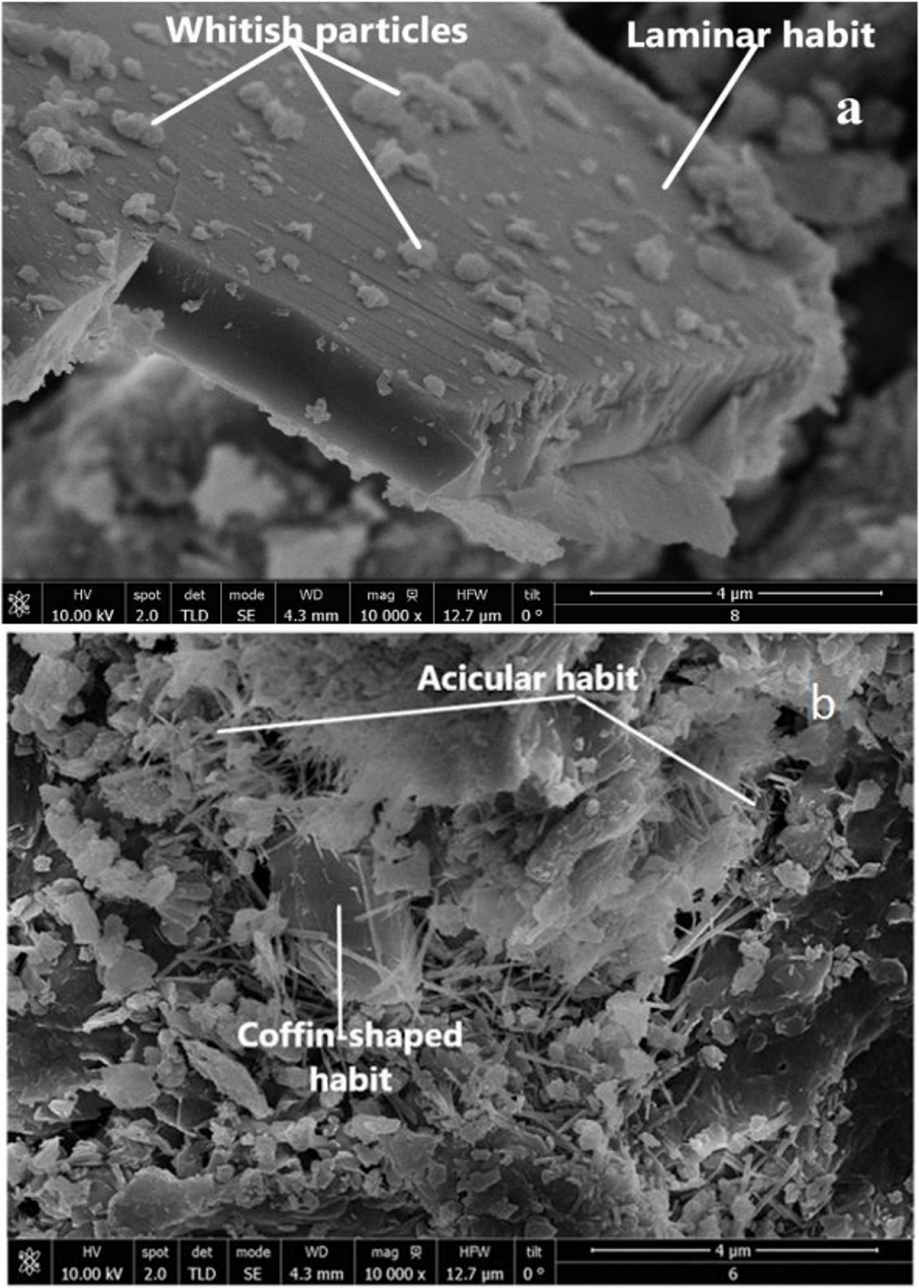
Micrographs of CLIM treated consecutively with: 2% DAP and 5 M urea solution (a), and 3.5% DAP and 5 M urea solution (b).

MOR crystals can be observed with their characteristic elongated morphology, some with a fibrous shape (as shown in Figure 5) and others acicular (as observed more clearly in Figure 6b) [45–46]. HEU crystals with laminar habit can also be distinguished, the morphology of which is associated with the presence of potassium [43–44].

The SEM images (Figure 6) of the modified materials (CLIM_f2-U5M_ and CLIM_f3.5-U5M_) show a cleaner surface as a consequence of zeolitic mineral processing in solutions. Note the laminar (Figure 6a) and coffin-shaped (Figure 6b) crystals of HEU, as well as the presence of agglomerates of elongated crystals with acicular to fibrous habit of MOR (Figure 6b) [43,46]. According to [15,47–48], the laminar habit is associated with K^+^ cations, while the coffin-shaped habit is associated with the predominance of both Ca^2+^ and K^+^ cations. This corresponds to the elemental composition (Table 1) of this natural zeolite (CLIM).

The images obtained from CLIM_f2-U5M_ (Figure 6a) show small bright particles on the surface of the HEU crystal, which may be related to the presence of urea. This is consistent with the previously stated, that is, that urea could be retained on the surface of this material. It is also consistent with the observed decrease in textural parameter values (Table 2). Like all zeolitic minerals, this natural zeolite possesses both porosity and mesoporosity. In this regard, note in the SEM images the spacing between the crystals of the zeolitic phases. It leads to diffusion of DAP and urea through them (porous and mesoporous) to the interior of mineral particles, which promotes their distribution over the material surface and their interaction with HEU and MOR crystals.

**Figure 7:**
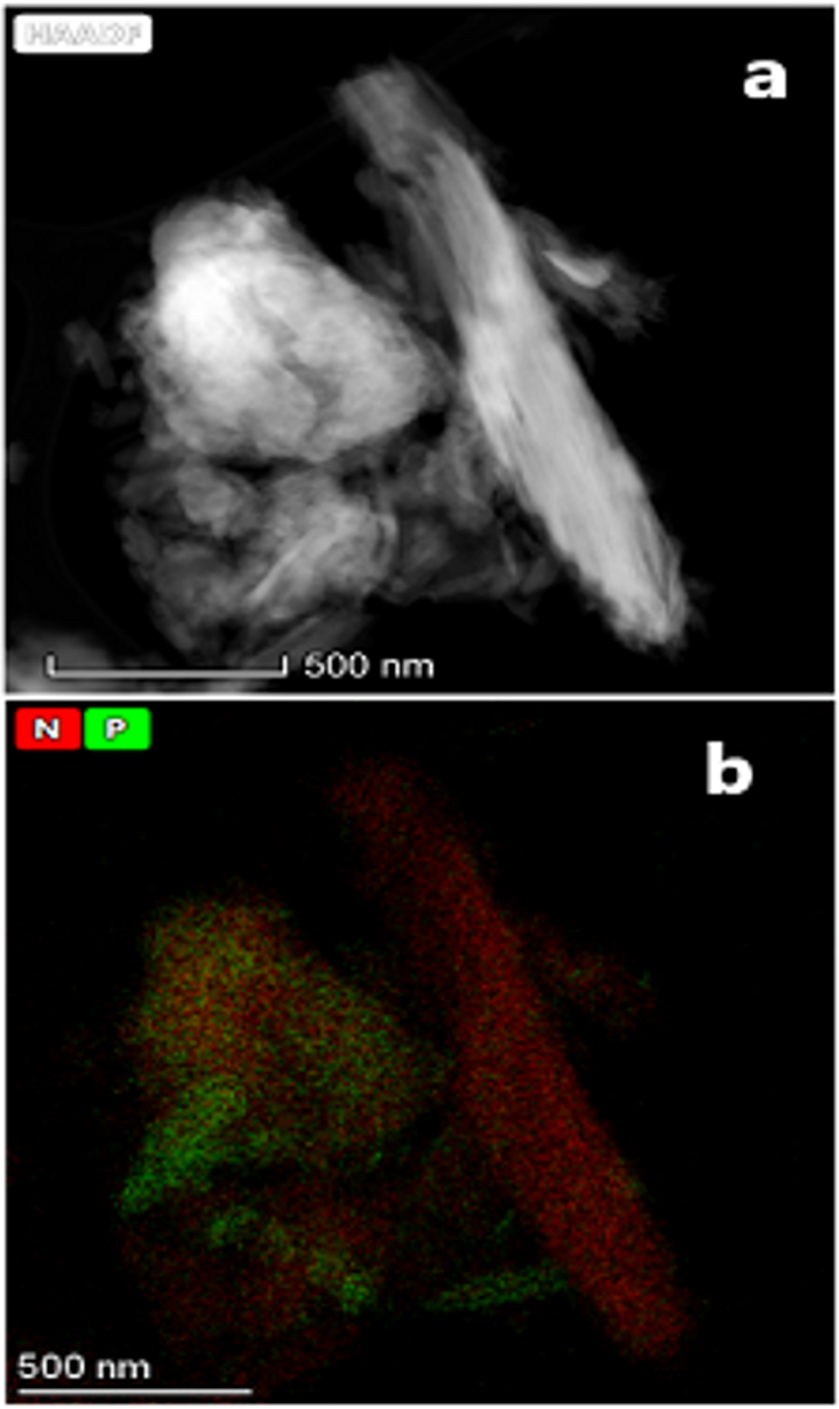
STEM micrographs of the CLIM_f2-U5M_ material (a) showing the combined elemental mapping for nitrogen and phosphorus (b).

**Figure 8:**
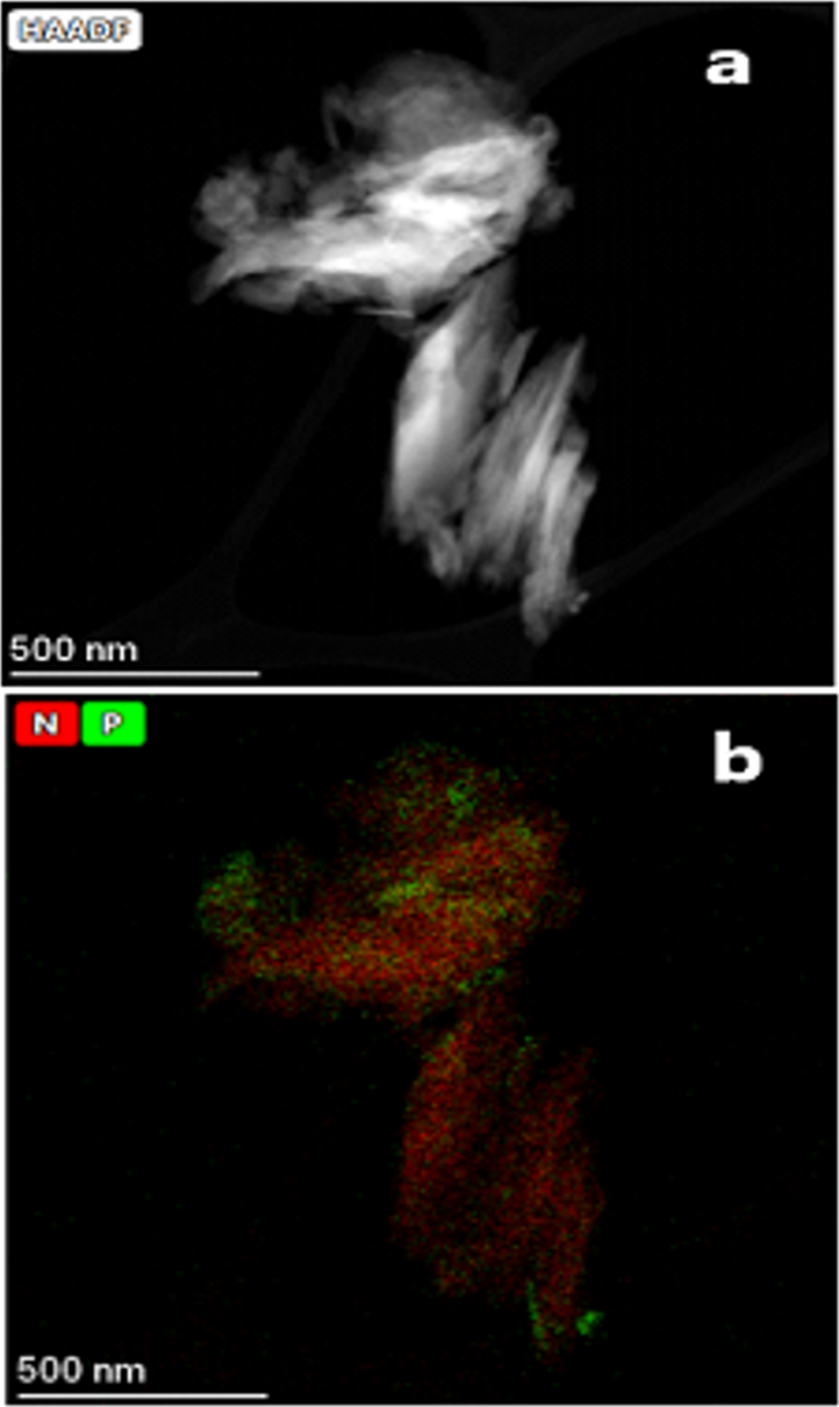
STEM micrographs of CLIM_f3.5-U5M_ material (a) showing the combined elemental mapping for nitrogen and phosphorus (b).

The STEM micrographs shown in Figure 7 and Figure 8, display the elemental mapping of nitrogen and phosphorus distributed on samples sequentially treated with DAP and urea (CLIM_f-U_). The combined mapping of nitrogen (red) and phosphorus (green) shows a homogeneous distribution of nutrients in the engineered materials and indicates the prevalence of nitrogen over phosphorus. These results validate the data obtained by FTIR, SEM, and adsorption isotherms, according to which urea is deposited on the outer surface of the zeolitic particles modified in a first stage with DAP.

### Pot culture trials

**Figure 9:**
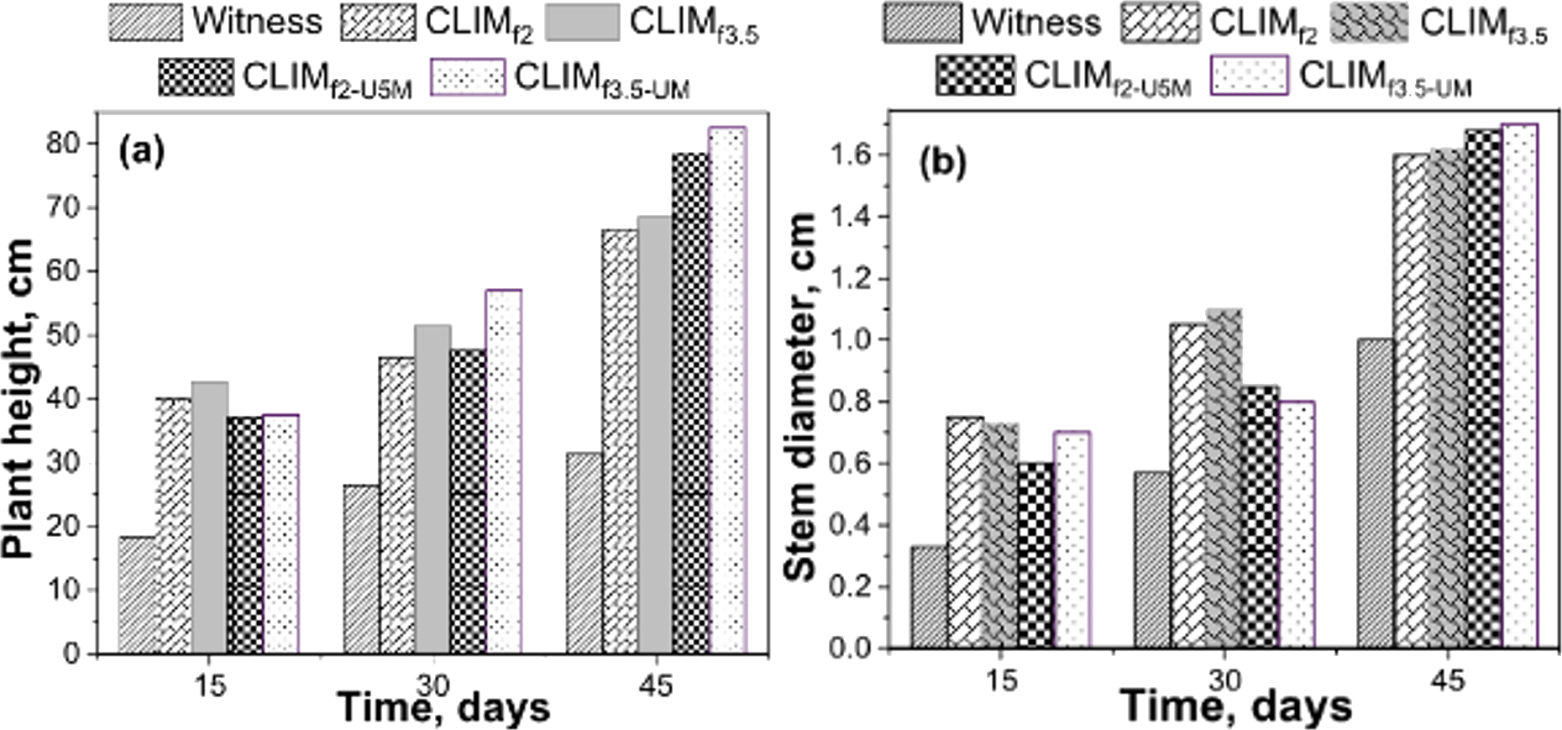
Average height (a) and stem diameter (b) values of maize plants recorded at 15, 30 and 45 days after planting. The maximum elevation (on the *Y*-axis) of each bar corresponds to the height and stem diameter of maize plants. These values correspond only to those recorded at 15, 30, and 45 days after planting; thus, they are grouped according to these times indicated on the *X*-axis.

Figure 9 shows the behaviour of controlled experimental parameters, namely, stem diameter and height of maize plants, during the 45 days of the study. In addition to materials sequentially modified with DAP and urea (CLIM_f2-U5M_ and CLIM_f3.5-U5M_), materials modified with DAP alone (CLIM_f2_ and CLIM_f3.5_) were also included to facilitate the analysis. Throughout the whole study, the values of the sample parameters of these materials were significantly higher than those of the control, indicating the great potential of these developed materials for use in plant cultivations. After the first 15 days, the materials modified with DAP alone showed the highest average values of stem height and diameter of cultivated plants.

However, on days 30–45 of the experiments, notable changes were observed; height and stem diameter of plants fertilized with CLIM_f2-U5M_ and CLIM_f3.5-U5M_ materials were larger than in those materials modified with DAP alone. This behaviour is consistent with the proposed surface coating mechanism of urea, where it acts as a physical barrier to limit the disproportionate release of nutrients.

These results validate that there is indeed an interaction between urea and zeolitic support at the surface level, where urea can be found coating the surface of the material. This coating becomes more permeable over time, facilitating the dissolution of urea and, therefore, the release of PO_4_^3−^ and NH_4_^+^ ions as well as other essential species (K^+^) provided by the mineral matrix. In this way, the delivery of nutrients to the soil in dissolved form for plant nutrition throughout the crop cycle is retarded. Surface-dissolved urea provides a significant input of nitrogen, which is essential mainly during the early stages of crop development. This scenario could lead to impeded diffusion and consequent slow release of nutrients from the zeolitic material into the soil solution. This late delivery of nutrients is consistent with the concept of slow or controlled release and increases the possibility of developing materials that adequately meet the requirements of crop nutrition and environmentally friendly agricultural activity.

## Conclusion

An exhaustive study was carried out on a zeolitic mineral consisting mainly of a mixture of two zeolites, mordenite (MOR) and clinoptilolite-heulandite (HEU), modified with aqueous solutions of ammonium hydrogenphosphate (DAP) and urea to develop materials carrying essential nutrients (N, P, K) and other elements (Si) important for agricultural crops. The presence of phosphate ions adsorbed on the zeolitic support, ammonium cations mainly exchanged in both zeolite phases, and urea arranged as a layer that covers the material and interacts with the zeolitic frameworks were confirmed by using FTIR spectroscopy, XRD, nitrogen adsorption measurements, SEM, STEM and other research methods, as well as culture tests.

The complexity of the zeolitic support (a mixture of MOR and HEU), compared to other similar materials developed on simpler zeolitic supports, such as those containing only HEU-type zeolite, leads to the appearance of a variety of types of applied compounds, which is evident from the change in the position and intensity of the bands in the FTIR spectra. Among all NH_4_^+^ bands, the band at 1402 cm^−1^ was the most intense, while for Cuban CLI from the Tasajeras deposit (also modified with DAP) it was located at 1540 cm^−1^. This difference is related to the higher NH_4_^+^ content in the MOR with respect to the HEU phase. Consistent with this, differences in the degree of band shift of the amino group of urea upon its interaction with the frameworks of these two different zeolites are also observed. The performed treatments did not affect the structural (as evidenced by XRD) and other qualities of these zeolites, preserving their porous structure, ion exchange and adsorption properties for reversible water retention, and slow release of nutrients (NH_4_^+^, PO_4_^3−^, etc.). This arrangement of urea affects the surface area of the materials, which is in agreement with SEM observations and elemental mapping (N and P) by STEM. Maize crop studies have demonstrated the great potential of zeolitic materials obtained by sequential modification with DAP and urea for plant development. Their composition allows them to provide nutrients in the required amounts throughout the crop cycle and is adequate to the concept of slow or controlled release. The use of zeolitic minerals in agriculture allows one to obtain low-cost, eco-efficient materials for massive application, with rational use of nutrients and agroecological benefits.

## Experimental

### Materials and methods

In this work, a zeolitic mineral from the San Andrés deposit in Holguín, Cuba, with a particle size class of +1–4 mm was used, which was supplied by the Industry Geominera-Holguín UEB, Cuba. This material basically is a mixture of MOR and HEU-type zeolites [49–50]; here, it will be referred to as CLIM or natural zeolite.

CLIM was first treated in solutions with variable amounts (2.0, 3.5, and 7.0%) of ammonium hydrogenphosphate (DAP with 18% N and 46% P) for 24 h, and then with aqueous solutions of urea with different molar concentrations (0.5, 1.0, 2.0, and 5.0 mol/L) and contact time (4, 8, 12, and 24 h) using a solid/liquid ratio of 1 g/10 mL and agitation on a shaker with horizontal movement. The treatments were applied following a procedure similar to that described in [13–14].

### Characterization

The elemental composition of CLIM and modified materials resulting from the applied treatments was determined by X-ray fluorescence analysis (XRF), with the exception of nitrogen, which was determined by N elemental analysis and the Kjeldahl method. XRF analysis was performed on an energy-dispersive spectrometer (Bruker Micro-XRF M4 Tornado, Nano GmbH) using tablets/briquettes of materials prepared for this purpose as described in [15]. For elemental analysis of N, a Leco elemental analyzer model CHNS-932 was used, for which 6 mg of sample were used, dried before at 60 °C and pulverized in a mortar. For the determination of N content by Kjeldahl, samples were dissolved by hydrothermal treatments with HF, HClO_4_, and HCl, followed by a procedure similar to that described in [15].

In addition, the samples were examined by Fourier-transform infrared spectroscopy (FTIR), X-ray diffraction (XRD), scanning electron microscopy (SEM), scanning transmission electron microscopy (STEM), and adsorption measurements based on N_2_ adsorption isotherms. FTIR spectra were recorded on a Bruker Tensor 27 IR spectrophotometer. The KBr tablet formation method was used with a KBr/sample ratio of 100:1. Powder XRD patterns were obtained on a Philips Xpert MPD diffractometer in the range from 2° to 60° using copper radiation (λ = 1.5406 Å), a speed of 2°/min, and a step of 0.05 s. For SEM studies, an FEI Nova Nano SEM 450 electron microscope was used; prior to measurements, samples were adhered to supports to coat them with a gold layer. For TEM analysis, samples were deposited on a 200-mesh copper grid with carbon membrane. Images were observed on a Talos F200X ThermoScientific microscope at 44000× magnification in S-MET mode, applying an accelerating voltage of 200 kV and using a high-angle annular dark-field detector. N_2_ adsorption isotherms at 77 K were obtained on a Micromeritics ASAP 2020 V4.04 (V4.04 H) instrument. Prior to adsorption measurements, the samples underwent vacuum thermal degassing for 8 h at 150 °C.

In order to obtain information on the fertilizing potential of the developed zeolitic materials, potted plant trials were carried out over a period of 45 days. For this purpose, black nylon bags were used, in which 700 g of a red ferrallitic soil and 27 g of developed material were added and mixed. Then, corn seeds were sown, making four replicates per treatment in a randomized block distribution. Red ferrallitic soil was used as a control for comparative purposes.

## Data Availability

Data generated and analyzed during this study is available from the corresponding author upon reasonable request.

## References

[1] Mariano E, de Sant Ana Filho C R, Bortoletto-Santos R, Bendassolli J A, Trivelin P C O (2019). Atmos Environ.

[2] Fincheira P, Hoffmann N, Tortella G, Ruiz A, Cornejo P, Diez M C, Seabra A B, Benavides-Mendoza A, Rubilar O (2023). Nanomaterials.

[3] Soltys L M, Mironyuk I F, Tatarchuk T R, Tsinurchyn V I (2020). Phys Chem Solid State.

[4] Mondal M, Biswas B, Garai S, Sarkar S, Banerjee H, Brahmachari K, Bandyopadhyay P K, Maitra S, Brestic M, Skalicky M (2021). Agronomy (Basel, Switz).

[5] Wise W S, Colella C (2013). Handbook of natural zeolites.

[6] Abdul Majid S, Ahmad Mir M, Mir J M (2018). J Chin Adv Mater Soc.

[7] Park M, Kim J S, Choi C L, Kim J-E, Heo N H, Komarneni S, Choi J (2005). J Controlled Release.

[8] Maghsoodi M R, Najafi N, Reyhanitabar A, Oustan S (2025). J Soil Sci Plant Nutr.

[9] Maghsoodi M R, Najafi N, Reyhanitabar A, Oustan S (2020). Geoderma.

[10] Conversa G, Pacifico S, La Rotonda P, Lazzizera C, Bonasia A, Elia A (2024). Eur J Agron.

[11] Choo L N L K, Ahmed O H, Talib S A A, Ghani M Z A, Sekot S (2020). Agronomy (Basel, Switz).

[12] Ruiz-Aguilar M Y, Aguirre-Uribe L A, Ramírez-Barrón S N, del Carmen Pérez-Luna Y, Castro-del Ángel E, Hernández-Juárez A (2024). Rev Mex Agroecosistemas.

[13] Rodríguez Fuentes G, Rivero González L, Rodríguez Iznaga I, Rivero Robaina E L (2023). Procedimiento para la obtención de sustratos y fertilizantes zeoliticos de liberación controlada y métodos de tratamiento de las plantas. Cuba. Pat. Appl..

[14] Rodríguez Fuentes G, Rivero González L, Rodríguez Iznaga I, Rivero Robaina E L (2021). Procedure to obtain substrates and controlled-release zeolitic fertilizers and plant treatment methods. Canada. Pat. Appl..

[15] de la Nuez Pantoja E Y, Iznaga I R, Fuentes G R, Petranovskii V, García A M, Gámez J J C, Jiménez D G, Cauqui M Á, Rivero González L A, García O C (2024). J Inorg Organomet Polym Mater.

[16] Rodríguez Iznaga I, Shelyapina M G, Petranovskii V (2022). Minerals (Basel, Switz).

[17] Rodríguez Fuentes G, Rodríguez-Iznaga I, Murrieta Rico F N, Antúnes García J, Petranovskii V (2025). Are NEREA®zeolitic nanostructured materials equal to NPK fertilizers mixed with natural zeolite?. Advancements in zeolites and micro-meso porous hierarchical materials.

[18] Zecchina A, Marchese L, Bordiga S, Pazè C, Gianotti E (1997). J Phys Chem B.

[19] Bučko T, Hafner J, Benco L (2004). J Chem Phys.

[20] Rodríguez-Iznaga I, Rodríguez-Fuentes G, Benítez-Aguilar A (2000). Microporous Mesoporous Mater.

[21] Rodríguez-Iznaga I, Rodríguez-Fuentes G, Petranovskii V (2018). Microporous Mesoporous Mater.

[22] Yadav V, Kumar L, Saini N, Yadav M, Singh N, Murugasen V, Varathan E (2023). Water, Air, Soil Pollut.

[23] Adriano A, Cornejo M H, Baykara H, Ludeña E V, Brito J L (2022). Materials.

[24] Xu D, Qin W, Zhang X, Liu Y (2025). ACS ES&T Water.

[25] Wei M, Tang X, Wang Y, Bai X, Wang X, Wang X, Wang P, Fang X, Li J, Yang J (2024). Sep Purif Technol.

[26] Bonelli B, Armandi M, Areán C O, Garrone E (2010). ChemPhysChem.

[27] Likhacheva A Y, Paukshtis E A, Seryotkin Y V, Shulgenko S G (2002). Phys Chem Miner.

[28] Gualtieri A F, Passaglia E (2006). Eur J Mineral.

[29] Liu B, Li S, Dai W, Liu F, Qin W, Wang M, Jia Y, Ma Z (2024). Chem Eng Sci.

[30] Putra R, Lestari W W, Susanto B H, Kadja G T M (2022). Energy Sources, Part A.

[31] Baran E J (2021). Metaloenzimas de plantas.

[32] Rodríguez-Fuentes G, Ruiz-Salvador A R, Mir M, Picazo O, Quintana G, Delgado M (1998). Microporous Mesoporous Mater.

[33] Mansouri N, Rikhtegar N, Panahi H A, Atabi F, Shahraki B K (2013). Environ Prot Eng.

[34] Samiei A, Ahmadi S H, Garmarudi A B, Badienejad M, de la Guardia M, Mateu D G (2025). Results Chem.

[35] Koyama K, Takeushi Y (1977). Z Kristallogr, Kristallgeom, Kristallphys, Kristallchem.

[36] Lanzafame P, Papanikolaou G, Barbera K, Centi G, Perathoner S (2019). J Energy Chem.

[37] Sun K, Su W, Fan F, Feng Z, Jansen T A P J, van Santen R A, Li C (2008). J Phys Chem A.

[38] Atkins P W (1992). The elements of physical chemistry.

[39] Byler D M, Gerasimowicz W V, Stockette V M, Eberl D D (1991). Microchem J.

[40] Millán G, Agosto F, Vázquez M, Botto L, Lombardi L, Juan L (2008). Cienc Invest Agrar.

[41] Cheah W-K, Sim Y-L, Yeoh F-Y (2016). Mater Chem Phys.

[42] Petrov O E, Ming D W, Mumpton F A (1995). Cation exchange in clinoptilolite: An X-ray powder diffraction analysis. Natural Zeolites 93: Occurrence, Properties, Use.

[43] Erdoğan B, Ergürhan O (2024). Clay Miner.

[44] Ateş E B (2022). J Turk Chem Soc, Sect B.

[45] Giordani M, Ballirano P, Pacella A, Meli M A, Roselli C, Di Lorenzo F, Fagiolino I, Mattioli M (2022). Minerals (Basel, Switz).

[46] Di Giuseppe D (2020). Crystals.

[47] Rodríguez-Fuentes G, Rodríguez-Iznaga I (2009). Rev Cubana Fis.

[48] Rodriguez-Fuentes G, de Ménorval L C, Reguera E, Chávez Rivas F (2008). Microporous Mesoporous Mater.

[49] Céspedes-Ortiz C, Rodríguez-Iznaga I, Petranovskii V, Rizo-Beyra R, Aguilera-Domínguez L (2011). Rev Cubana Quim.

[50] Costafreda J L, Martín D A (2021). Molecules.

